# Radionuclide Shunt Scintigraphy Technique in Diagnosing Ventriculoperitoneal Shunt Malfunction and Patency: A Case Study and Review of Literature

**DOI:** 10.7759/cureus.83184

**Published:** 2025-04-29

**Authors:** Zakir Chew, Daniel Loh, Adriel Leong, Hoi Yin Loi, Tseng Tsai Yeo

**Affiliations:** 1 Department of Surgery, Division of Neurosurgery, National University Hospital Singapore, Singapore, SGP; 2 Department of Diagnostic Imaging, National University Hospital Singapore, Singapore, SGP; 3 Department of Nuclear Medicine, National University Hospital Singapore, Singapore, SGP

**Keywords:** hydrocephalus, radionuclide shunt scintigraphy, thermal transfer, ultrasound and doppler techniques, ventriculoperitoneal shunt malfunction

## Abstract

Although cerebrospinal fluid (CSF) diversion through ventriculoperitoneal shunt insertion is a conventional treatment for hydrocephalus, shunt malfunction is a common complication, making its diagnosis critical. We report a patient with a background of congenital hydrocephalus and an in situ ventriculoperitoneal shunt (VPS) exhibiting worsening papilledema with raised lumbar puncture opening pressures, indicating a possible VPS malfunction. A review of the current diagnostic methods for VPS malfunction, focusing on radionuclide scintigraphy, was conducted in this case review. VPS radionuclide scintigraphy was successfully performed in our patient using intrathecal administration of Tc-99m diethylene-triamine-penta-acetic acid (DTPA) into the CSF shunt reservoir, facilitating tracer visualization. A review of the patient’s case notes and subsequent literature review identified several methods for diagnosing VPS malfunction to determine the correct shunt malfunction segment and respective corrective measures. VPS radionuclide scintigraphy in our patient confirmed a patent distal peritoneal catheter, although a transient proximal ventricular catheter blockage and malfunction remained a possibility. The literature review highlighted successful worldwide applications of substance dilution, thermal transfer, ultrasound, and Doppler techniques for diagnosing VPS malfunction with radionuclide shunt scintigraphy. Evaluating VPS function comprises various methods and is significantly important to prevent adverse consequences for the patient, allowing prompt revision surgery directed at the site of obstruction or malfunction. Hence, VPS malfunction diagnosis is vital for leading appropriate corrective measures, with radionuclide shunt scintigraphy functioning as a non-invasive method.

## Introduction

Hydrocephalus is the most common pediatric neurological disorder, characterized by the pathologic accumulation of cerebrospinal fluid (CSF), causing active distension of the ventricular system due to the inadequate flow of CSF from its production to absorption sites [1]. This harmful excess fluid increases intracranial pressure, leading to age-dependent symptoms that range from a rapid increase in head size or an abnormally large head size in infants to common symptoms of headaches, drowsiness, nausea or vomiting, vision problems, ataxia, impaired mentation, and loss of bladder control or frequent urination [2].

Untreated hydrocephalus may lead to macrocephaly, cognitive dysfunction, or even death [1]. While childhood hydrocephalus represents a chronic disease, adult-onset hydrocephalus may result from infection, trauma, tumor-related obstruction, or idiopathic causes such as normal pressure hydrocephalus [1].

Once diagnosed, hydrocephalus requires CSF diversion procedures. Pediatric hydrocephalus is often treated with endoscopic third ventriculostomy, ventriculoperitoneal shunts (VPS), or temporizing external ventricular drains. Long-term complications from these procedures include infections and mechanical malfunctions, notably progressive occlusion of the third ventriculostomy stoma or shunt blockages. 

Diagnosing shunt blockages is of significant importance, as VPS malfunction is associated with increased morbidity and mortality [3]. Moreover, neuroimaging may not consistently correlate with clinical outcomes, as ventricular size may maintain normality despite VPS malfunction, and slight changes in ventricular size are also difficult to detect [4].

Multiple techniques have existed to aid in the diagnosis of VPS malfunction, with VPS radionuclide scintigraphy being successfully used worldwide to determine which portion of the shunt is non-functioning [4,5]. The technique has been described as early as the 1970s, which makes use of a small volume of radionuclide being injected into the shunt reservoir, followed by imaging for visualization that can determine the segment that has obstruction or if over-drainage occurred [5].

This case report and review aim to assess a relatively non-invasive method for functionally evaluating a VPS in a presented case while reviewing the current literature on shunt malfunction diagnosis.

## Case presentation

**Figure 1:**
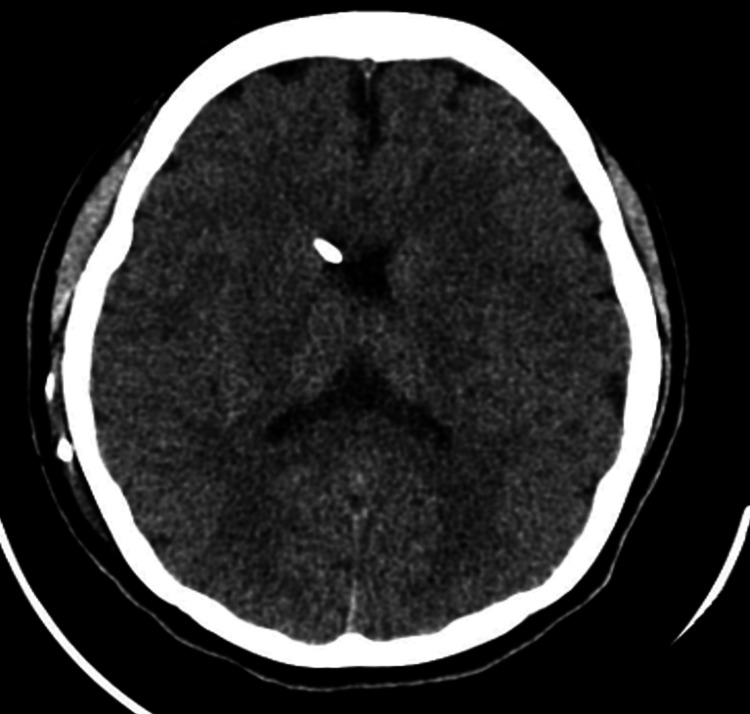
CT brain (axial view) showing ventricles of normal size with a VPS in situ. CT: computed tomography; VPS: ventriculoperitoneal shunt

We present the case of a 17-year-old girl with a history of congenital hydrocephalus with a possible shunt malfunction. She required a shunt revision once at 12 years of age, also due to a previous shunt malfunction in 2019, initially presenting with right-sided neck swelling, and X-ray imaging of the neck showed a fractured VP shunt. A medium-pressure non-programmable shunt (Medtronic, Minnesota, USA) was inserted at that time, leaving the fractured ends of the shunt behind. She recently presented again, this time with worsening papilledema (over three months) during a routine ophthalmology review, in which she was on follow-up for visual field monitoring for her pituitary mass since 2020. Reservoir filling was intact, with a computed tomography (CT) of the brain revealing no signs of hydrocephalus, as seen in Figure 1.

**Figure 2:**
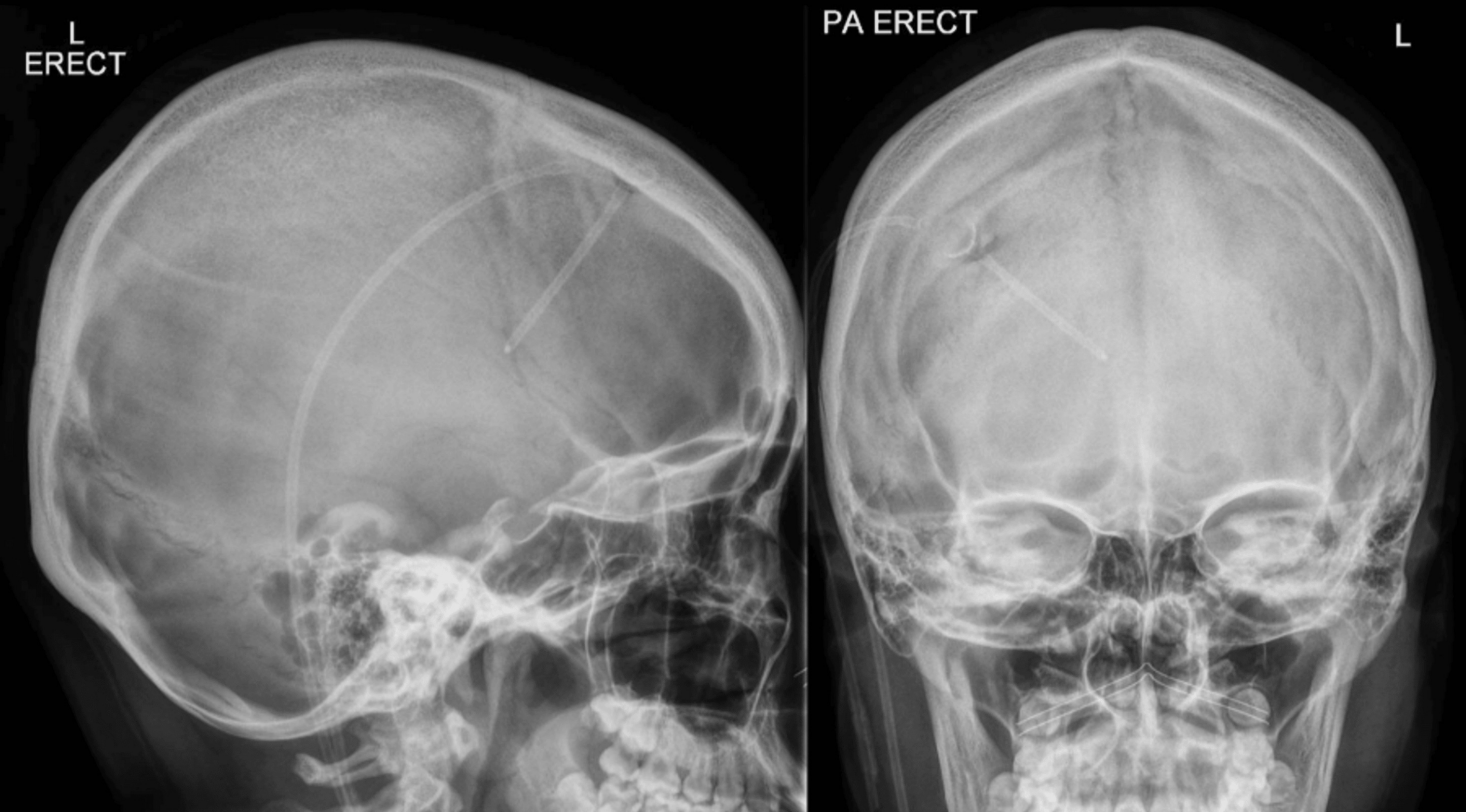
X-ray skull imaging in anteroposterior and lateral views.

**Figure 3:**
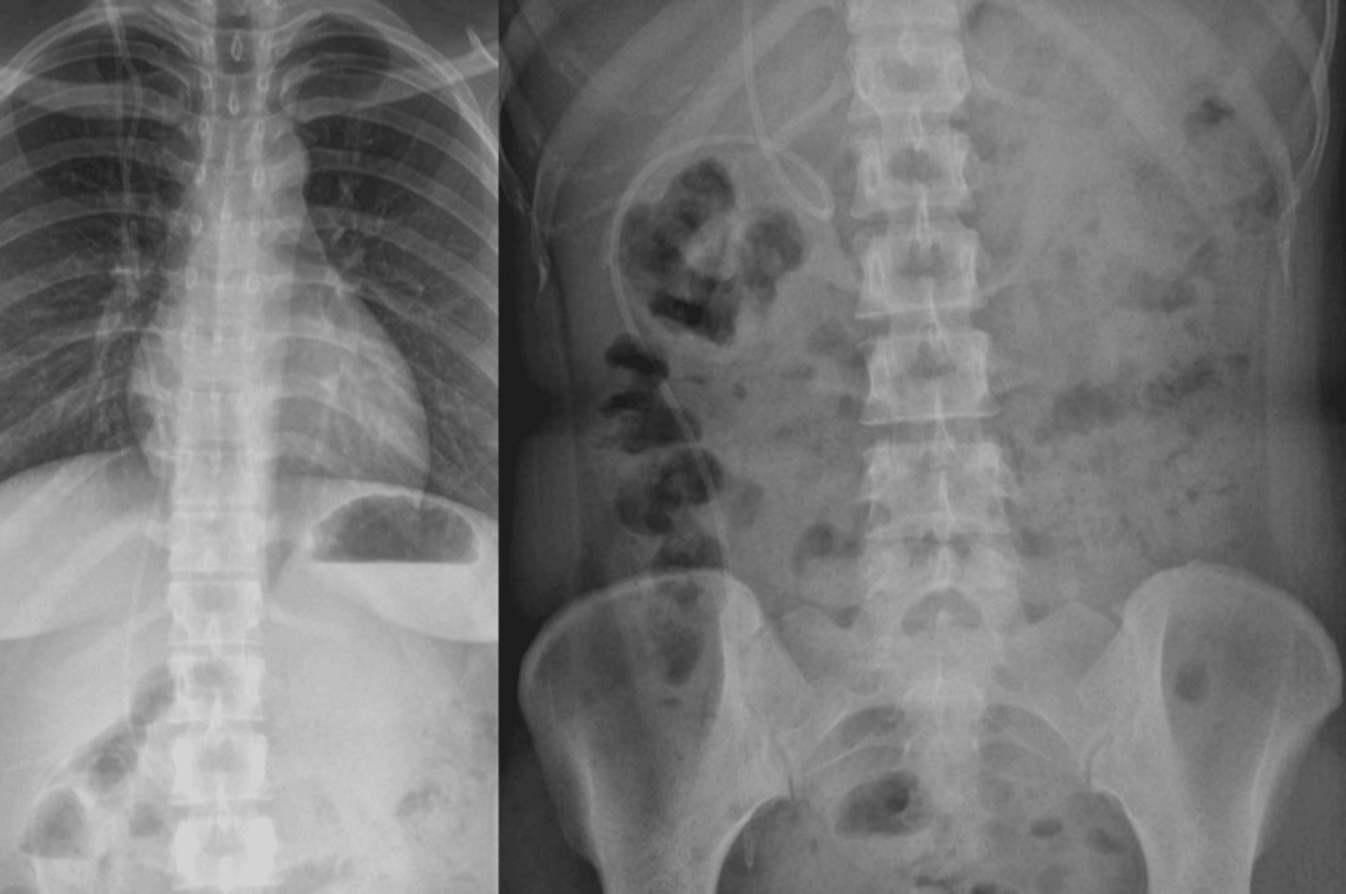
X-ray chest and abdomen to visualize the distal end of the VPS. VPS: ventriculoperitoneal shunt

Shunt series XR imaging did not show any fracture or kinks in the revised VP shunt seen in Figures 2, 3. A lumbar puncture was performed to obtain CSF samples that were clear and unremarkable, although an elevated opening pressure of 23 cm H₂O was elicited, raising questions regarding shunt patency.

**Figure 4:**
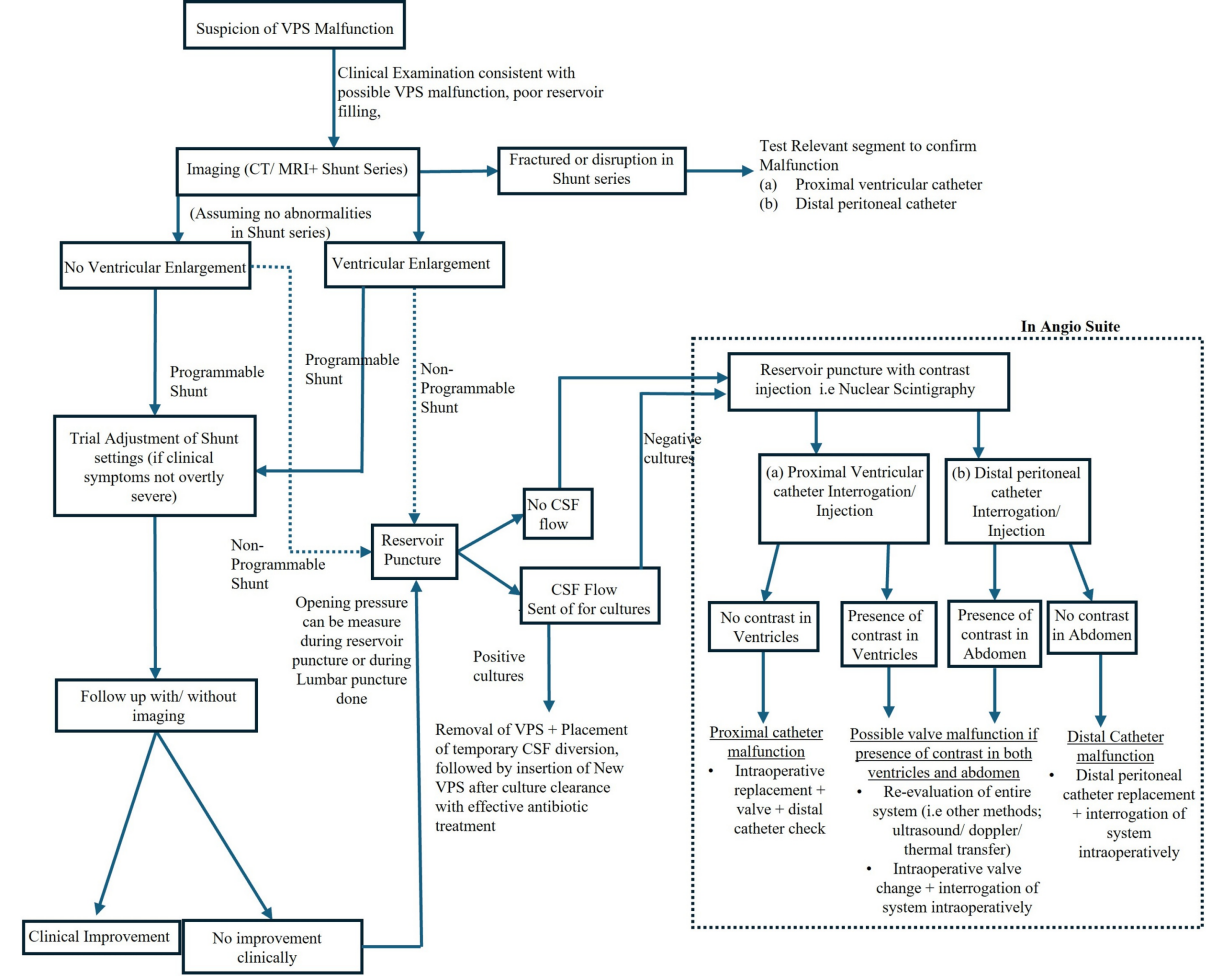
Proposed algorithm to determine ventriculoperitoneal shunt malfunction. The image is created by the authors, adapted from the literature review of Broggi et al (2009), Rot et al (2020), and Kharkar et al (2022) [4,6,7] VPS: ventriculoperitoneal shunt; CT: computed tomography; MRI: magnetic resonance imaging; CSF: cerebrospinal fluid

**Figure 5:**
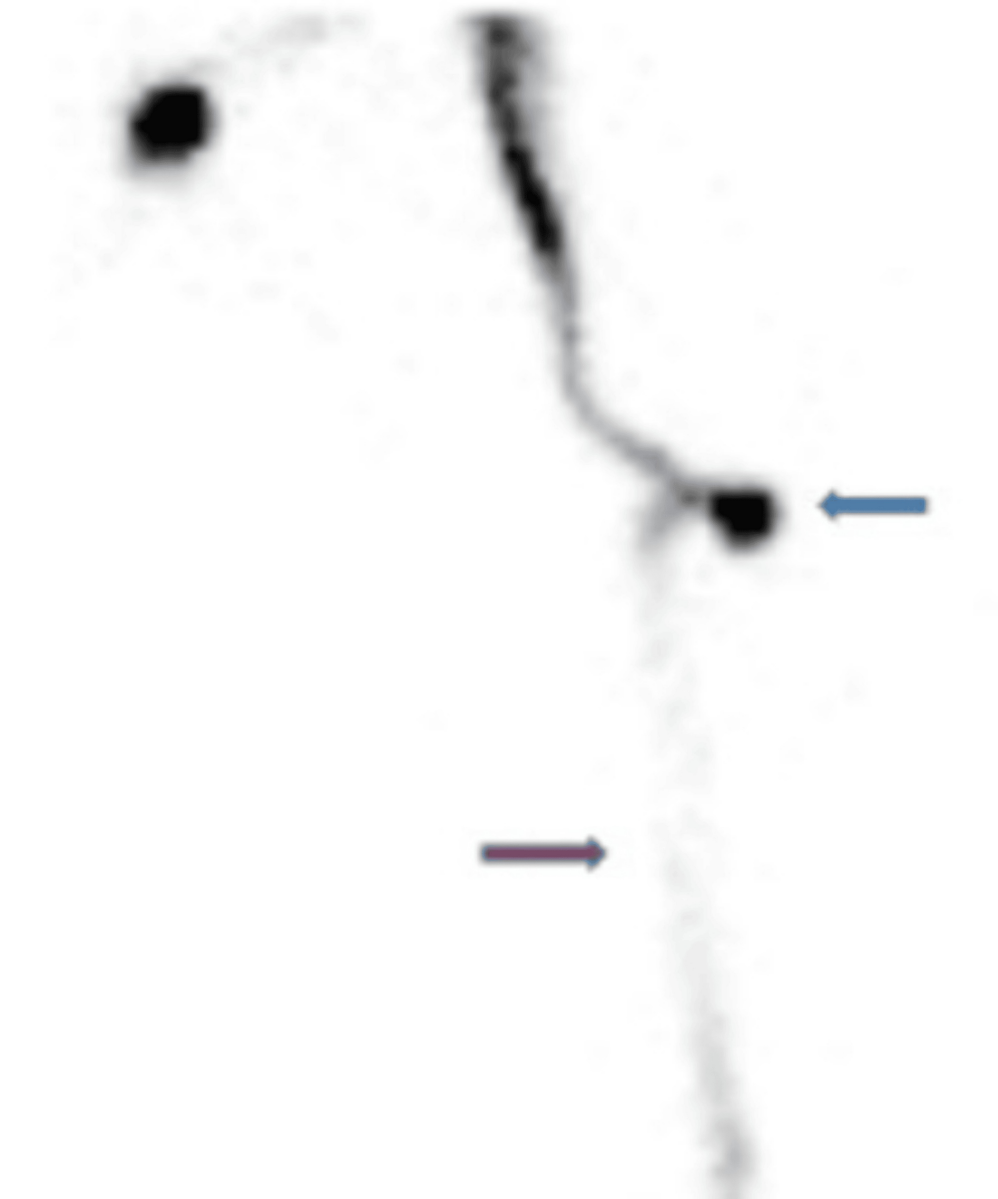
Intrathecal administration of Tc-99m DTPA radionuclide through puncture in the shunt reservoir (blue arrow) showing visualization of radiotracer followed by downstream flow down the distal catheter (red arrow). Tc-99m DTPA: technetium-99m diethylene-triamine-penta-acetic acid

**Figure 6:**
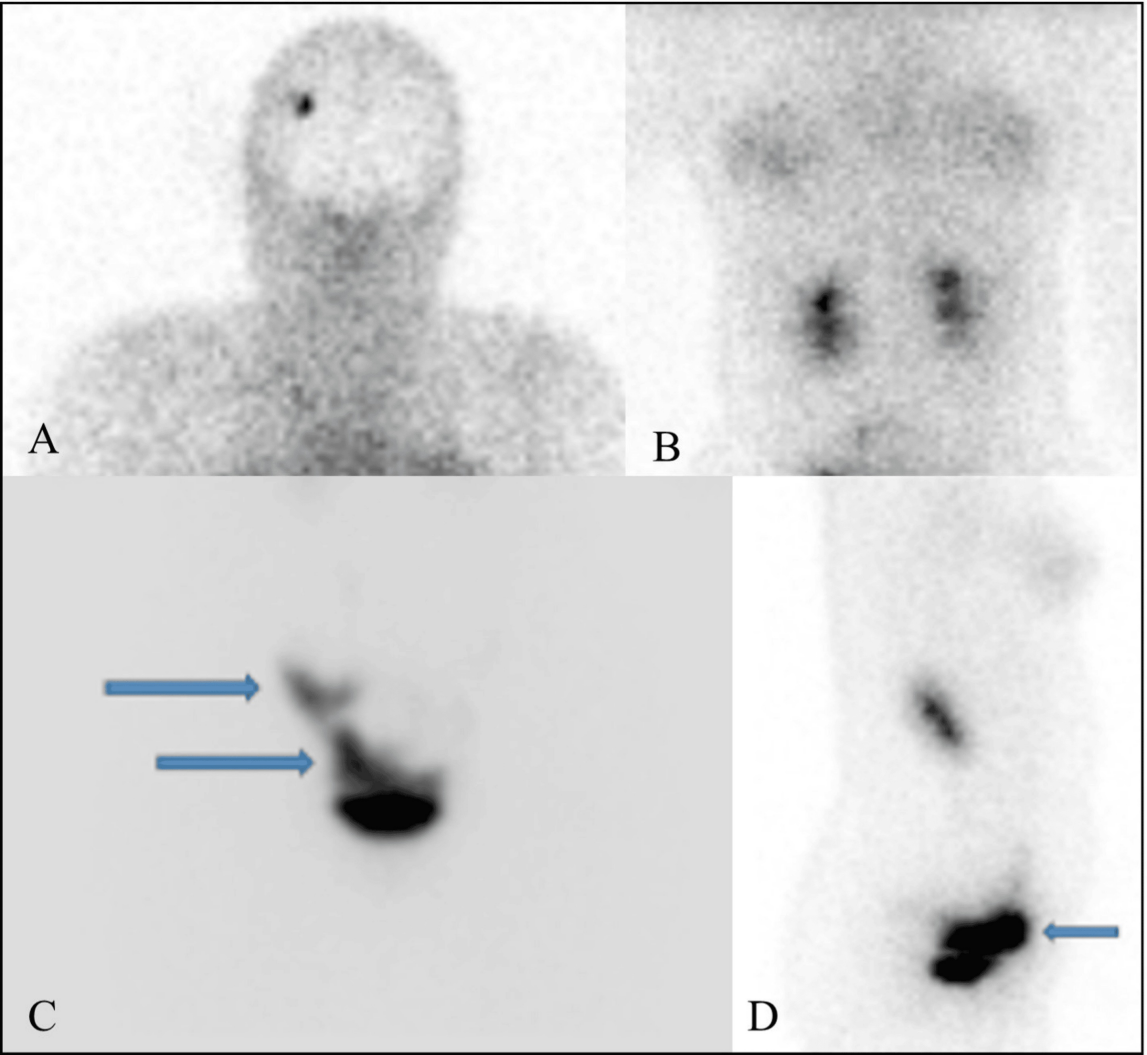
Findings. A-C: delayed static anterior views of the head to pelvis showing visualization of radiotracer activity with accumulation in the pelvic region (blue arrows) upon completion of the dynamic study; D: lateral view showing radiotracer activity within the expected pelvic region (blue arrows).

She was started on a trial of oral furosemide, as her glucose-6-phosphate dehydrogenase deficiency (G6PD) prevented the use of acetazolamide. Furosemide was initially dosed at 40 mg twice a day for 42 days and subsequently reduced to 40 mg once daily for a week before the medication was stopped in view of improvement in her papilledema during her subsequent eye reviews. A literature review was conducted to determine the patency of VPS, yielding several perspectives. Moreover, an algorithm adapted from Kharkar et al. (2009), Broggi et al. (2020), and Rot et al. (2022), along with current institutional guidelines, was employed, as depicted in Figure 4 [4,6,7]. The functional status of the shunt via radionuclide shunt scintigraphy was evaluated. VPS radionuclide scintigraphy was performed with 18.2 megabecquerel (MBq) intrathecal administration of Tc-99m diethylene-triamine-penta-acetic acid (DTPA) into the CSF shunt reservoir. The tracer was immediately visualized upon injection in delayed static views in the subsequent dynamic study (10s per frame for 10 minutes), as seen in Figures 5, 6, with eventual excretion through reabsorption of the tracer within the peritoneal cavity into the bloodstream and out through the urogenital system. Radionuclide scintigraphy, together with the various other methods such as X-rays (XR), CT scans, and lumbar puncture, coupled with our patient's clinical improvement, helped ascertain that the VPS was working. She was discharged with further follow-up, without any surgical revision of her VPS. 

## Discussion

By employing our adapted algorithm, although imaging did not reveal hydrocephalus, the raised opening pressure of 23 cm H₂O during lumbar puncture and improvement of symptoms with furosemide suggested the possibility of shunt malfunction. An X-ray shunt series was conducted, indicating no fracture or disruption in the shunt from the ventricular to the peritoneal end. Eventually, we performed radionuclide scintigraphy to further evaluate the VPS. While several methods to determine shunt patency, such as substrate dilution, were researched, these had not been extensively replicated and performed, and entailed multiple reservoir punctures in a young patient. Thus, they were not employed, and the decision to implement radionuclide scintigraphy was favored.

Radionuclide scintigraphy findings revealed that the distant shunt was patent and functional, although no contrast was delivered proximally, raising suspicion of a partially blocked ventricular catheter. 

Multiple techniques are available to evaluate the function of shunts, mainly through open surgery, an invasive method associated with risks of general anesthesia and surgical complications. Prior to considering invasive surgical intervention, we employed a radionuclide shunt scintigraphy method. After injecting the Tc-99m DTPA tracer, delayed static views from the head to the pelvis were obtained upon completing the dynamic study. The residual radiotracer was noted within the reservoir without hold-up activity along the expected course of the VPS. Intense radiotracer activity was observed within the expected pelvic region where the distal end of the shunt lies, implying a functional distal ventricular catheter.

Shunt malfunction is defined as a partial or complete blockage of the catheter or valve and can be caused by disconnection or fracture of the catheter. Partial blockage can occur in which blockage is intermittent or flow is not completely blocked in the VPS, in which aspiration of CSF from the shunt or injection of dye into the shunt is difficult [4]. There are various methods to diagnose a VPS malfunction, with possible malfunctions occurring in different compartments of a shunt. A shunt comprises a proximal end connected from the reservoir and valve to the ventricles, the valve itself, and a distal portion that connects the valve to the peritoneal area. Some non-invasive methods of checking for shunt malfunction include clinical examinations that may include a wide array of symptoms such as headache, nausea, vomiting, or vision problems. Valve reservoir palpation is routinely implemented to identify rapid re-expansion to exclude ventricular catheter obstruction. This approach is regarded as a simple and reliable means of assessing the function of the shunt [4].

After clinical examination, imaging such as a CT of the brain can be performed to determine the dimensions of ventricular distension, if present, or shunt series imaging. Imaging would indicate disconnections or breakages along the shunt, determining the integrity of the entire shunt system. However, it is important to recognize that false negatives concerning brain imaging may arise [8].

It is important to distinguish the precise malfunctioned portion of the shunt, whether due to obstruction or catheter fracture, as revision surgery may involve partial replacement of the proximal ventricular catheter, the valve, the distal peritoneal catheter, or replacement of the whole system. Once a clear shunt malfunction is diagnosed, surgical revision can determine the malfunctioning portion of the shunt. Diminished or absent CSF outflow from the proximal ventricular catheter may signify an obstructed ventricular catheter, while valve and distal peritoneal catheter malfunction can be determined with the aid of a fluid column in a connected manometer [5]. 

Substrate dilutions

Several methods have been employed, including one that leverages substance dilutions. The underlying mechanism of shunt dilution is that, in the occurrence of a shunt malfunction, the lack of free CSF flow through the shunt leads to a higher concentration of liquid in the shunt reservoir. For various types of shunts used, different flow rates of CSF may be obtained. As a result, in vitro experiments correlating baseline CSF flow with the introduction of substances such as sodium valproate (SV) or glucose are required, as described by Fu et al. (2021) and Zhang et al. (2017) [9,10]. One limitation is that it may necessitate multiple measurements with several reservoir punctures, increasing the risk of infections.

Otoacoustic emissions (OAE)

Another novel method, introduced by Sakka et al. (2016), utilizes otoacoustic emissions (OAE) to determine the functionality of VPS [11]. This method removes the need for valve puncturing, although the specific segment of shunt malfunction is undeterminable. OAE varies with increased intracranial pressure and functions as a non-invasive tool to detect shunt malfunctions compared to the traditional method that requires a shunt tap or injection performed by a neurosurgical team.

Thermal transfer

The majority of VPS patency tests were developed and conducted primarily on pediatric populations. For example, the thermal transfer method uses a thermistor at the distal end, cooling a portion proximally, followed by pumping the shunt [12]. Such earlier designs, based on thermal convection or flow evaluation using thermistors to measure CSF flow [12], have led to the development of novel specialized devices, including ShuntCheck® (NeuroDx Development, LLC, PA, USA) (with or without a micro-pumper), proving effective in detecting VPS malfunctions [13]. Such techniques employ the detection of cooled CSF through a temperature-sensing device, which detects temperature changes as the cooled CSF flows along the catheter, implying a patent and functional VPS. This technique could be employed in detecting VPS malfunctions if such devices are available in institutions.

Lumbar infusion test, ultrasound, and Doppler imaging techniques

Other methods that were also employed in pediatric populations include the lumbar infusion test by Schutz et al. (1983), the Doppler technique by Flitter et al. (1975), and the intraventricular injection of radioactive iodinated serum albumin (RISA) by Kagen et al. (1963) [14-16]. Kaplan et al. (2007) also employed Doppler ultrasound techniques, analyzing the waveform patterns generated from reservoir pumping to determine shunt patency [17].

Phase contrast magnetic resonance (MR) imaging

In addition to static CT or MR imaging, various phase-contrast MR imaging techniques, which detect flow rates in the ventricular system and determine shunt patency, have been reviewed by Korbecki et al. (2019). However, cases with very slow CSF flow could be mistaken for a non-functioning shunt [18]. Furthermore, this technique exhibits various technical limitations, as quantitative assessment is only possible in a single plane. Despite this, it provides a potential non-invasive method for detecting VPS malfunctions [18]. Employment of such techniques would require adequate imaging techniques and trained personnel for the interpretation of such CSF studies.

Radionuclide shunt scintigraphy

In our presenting case, we implemented the globally recognized and adopted technique of radionuclide shunt scintigraphy to determine the malfunctioning segment of the VPS. This method was first described by Di Chiro and Grove in 1966, using radionuclide isotracers followed by dynamic imaging [19]. This approach has been performed by several other researchers to determine partial or total blockages of shunt catheter segments, as well as which segment of the catheter has malfunctioned or has become blocked [6,7,11]. Radionuclide shunt scintigraphy has also been successfully implemented in patients with normal-pressure hydrocephalus [6].

If patients show no improvement of symptoms, other methods such as thermal flow transfer, ultrasound, or Doppler techniques, or substrate dilution could be employed, with surgical exploration and shunt revision being the definite approach to diagnose malfunction, as shown in Figure 4.

In our case study, our patient eventually exhibited clinical improvement, with reduced papilledema, including after discontinuing diuretic treatment (furosemide). There were initial concerns of a blocked ventricular catheter, given that the injection of tracer was met with resistance. However, given clinical improvement even after cessation of furosemide, we concluded that there could have been a transient partial blockage of the shunt that subsequently spontaneously resolved, which is a phenomenon that has been reported in the literature [20]. Nonetheless, radionuclide scintigraphy assisted in evaluating shunt functionality and patency. 

## Conclusions

Suspected VPS malfunction requires extensive tests, ranging from clinical examination findings and imaging to more invasive techniques, including shunt reservoir interrogations to determine the malfunctioning segment, if present. Prompt surgical intervention can then be undertaken depending on the segment that has malfunctioned. Evaluating shunt patency is important to prevent serious consequences for the patient, ensuring that revision surgery can be performed promptly and directed to the correct site of obstruction. Radionuclide shunt scintigraphy is a well-established method used by multiple institutions worldwide that can successfully verify the patency of VPS and diagnose VPS malfunctions.
